# Longitudinally continuous varying high-order cylindrical vector fields enabled by spin-decoupled metasurfaces

**DOI:** 10.1515/nanoph-2024-0008

**Published:** 2024-03-04

**Authors:** Xinye He, Hanlin Bao, Fei Zhang, Tongtong Kang, Mingbo Pu, Yan Chen, Yinghui Guo, Jintao Gong, Mingfeng Xu, Xiangang Luo

**Affiliations:** National Key Laboratory of Optical Field Manipulation Science and Technology, Chinese Academy of Sciences, Chengdu 610209, China; 74709 State Key Laboratory of Optical Technologies on Nano-Fabrication and Micro-Engineering, Institute of Optics and Electronics, Chinese Academy of Sciences, Chengdu 610209, China; College of Materials Sciences and Opto-Electronic Technology, University of Chinese Academy of Sciences, Beijing 100049, China; 74709 Research Center on Vector Optical Fields, Institute of Optics and Electronics, Chinese Academy of Sciences, Chengdu 610209, China

**Keywords:** metasurfaces, control of vector optical fields, asymmetric PSOIs

## Abstract

The manipulation of vector optical fields in three-dimensional (3D) space plays a vital role in both fundamental research and practical implementations of polarization optics. However, existing studies mostly focus on 3D vector optical fields with limited modes. Here, an approach of spin-decoupled spatial partitioning is proposed to generate complex 3D vector optical fields with a customizable number of modes on demand. The crosstalk among different modes is effectively suppressed by the decoupling capability of asymmetric photonic spin–orbit interactions (PSOIs) and the design of region displacement for opposite spin states. As a proof-of-concept demonstration, a metasurface is designed to generate longitudinally varying high-order cylindrical vector fields, ranging from the 2nd to the 10th order in even sequences along the propagation direction. The experimental results demonstrate the effectiveness and potential of our approach to enabling precise control of 3D vector optical fields with arbitrary mode combinations. This work holds promising applications in biophotonics, quantum optics, and communications.

## 1 Introduction

Polarization is one of the fundamental properties of light, and the precise control of the polarization distribution of vector optical fields has aroused growing interest in practical applications such as quantum information [1], super-resolution microscopy [2], and optical storage [3]. Particularly, generating high-order cylindrical vector fields with excellent characteristics possesses great research and application prospects in various fields. For example, the notable robustness of polarization singularities in atmospheric turbulence enhances the potential value of high-order cylindrical vector optical fields in free-space communication [4]. Furthermore, utilizing multiple states of the high-order vector optical field offers opportunities for information encoding/decoding [5]. Creating complex 3D vector optical fields is challenging for conventional optics, due to the limited ability of optical field manipulation. Moreover, traditional optical components typically treat polarization control as a globally uniform characteristic. Hence, it is necessary to develop innovative methods for polarization state control, to augment the capabilities and applications of vector optical fields.

In recent years, metasurfaces composed of subwavelength structures have attracted increasing attention, owing to their exotic capability to manipulate electromagnetic wave parameters, such as frequency, amplitude, polarization, and phase [6], [7], [8], [9], [10], [11], [12], [13], [14], [15], [16]. Furthermore, they also emerge as a new platform for generating and manipulating vector optical fields [17], [18], [19], [20]. Through the combination of the propagation phase [21], [22], [23], [24] and geometric phase [25], [26], [27], composite-phase metasurfaces enable the independent control of the two orthogonal polarization states, resulting in asymmetric photonic spin–orbit interactions (PSOIs) [28], [29], [30], [31], [32]. Notably, composite-phase metasurfaces have demonstrated great advantages in various fields, such as flat imaging [33], [34], vector visual cryptography [35], [36], orbital angular momentum detection [37], [38], and holography [39], [40], [41], [42].

Currently, research on vector field manipulation has made significant strides across multiple dimensions, progressively extending from two-dimensional (2D) to 3D space modulation [43], [44], [45], [46], [47], [48], [49]. On the one hand, the longitudinally varying polarization state adds a new dimension for longitudinal detection and volume laser machining [50], [51]. On the other hand, generating multi-order vector optical fields along the propagation direction opens up new possibilities for optical manipulation [52], due to the differences in the optical forces and motion behaviors of particles subjected to different modes of vector optical fields. However, the degree of freedom and flexibility in controlling 3D vector optical fields remain relatively limited in the majority of studies. For example, a universal all-dielectric metasurface platform can generate high-order vector fields [49], but the modulation is used to map the polarization state in several limited transverse planes. Moreover, a continuous variation of polarization states along the longitudinal direction can be achieved through the utilization of form-birefringent metasurface [18], but the topological charge is limited to 1st order and not expanded to higher orders due to its design structure. Consequently, the current studies encounter a challenge in producing longitudinally continuous multiple high-order light fields.

In this work, we propose an approach of spin-decoupled spatial partitioning to generate continuously varying high-order 3D vector optical fields with arbitrarily adjustable modes along the propagation direction. This region displacement for opposite spin states is designed to achieve the generation and transformation of an arbitrary-order cylindrical vector field and Bessel beam, as well as the conversion from a uniform scalar optical field to a 3D vector optical field. As a proof of concept, we demonstrate longitudinally varying high-order cylindrical vector fields with different orders ranging from the 2nd to the 10th along the propagation direction.

## 2 Design and method

### 2.1 Concept of 3D high-order cylindrical vector fields

**Figure 1:**
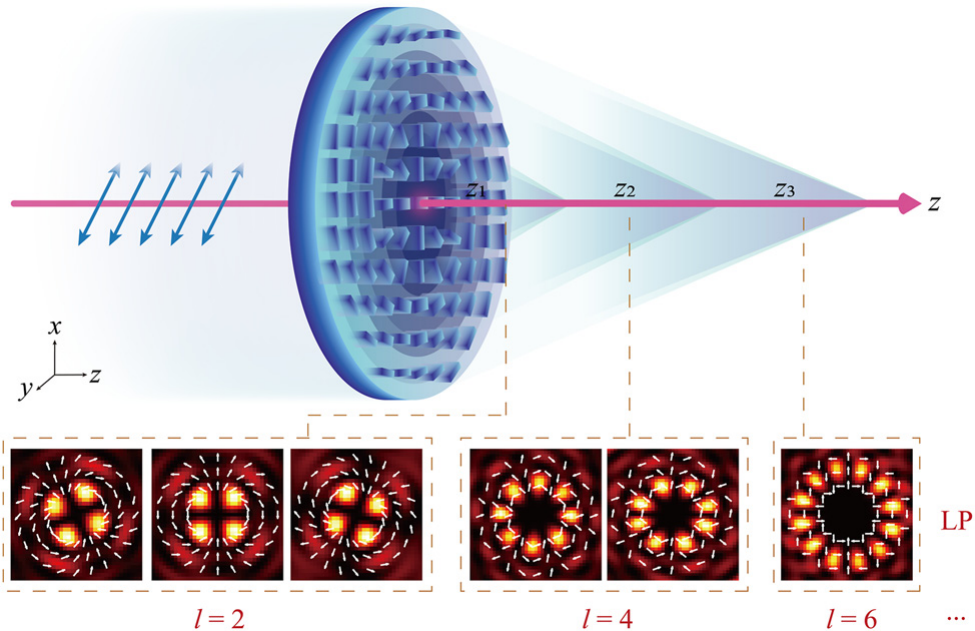
Schematic diagram of generating 3D high-order cylindrical vector fields via a single metasurface. The case shows the 2nd-order, 4th-order, and 6th-order light spot diagrams of the *x*-component under the illumination of *x*-LP light.

Figure 1 illustrates the synthesized high-order cylindrical vector fields using a region displacement design with opposite spin states. In this case, 2nd, 4th, and 6th-order optical fields continuously change along the propagation direction in regions *z*_1_, *z*_2_, and *z*_3_, respectively. Specifically, the variables *l* = 2, 4, and 6 indicate the order of cylindrical vector optical fields, with a detailed definition provided in Section 2.2. The linearly polarized (LP) incident light is focused with a long focal depth and the vector optical fields of different orders correspond to the predesigned longitudinal region. The ring-shaped regions of left circularly polarized (LCP) and right circularly polarized (RCP) light manipulated by the designed metasurface are linearly related to the longitudinal length of the modes to be generated according to the geometrical relationship. In particular, the focusing Bessel beam cone angles for the LCP and RCP lights are different, resulting in distinct propagation constants for the two opposite spin states of light along the propagation direction. This difference causes a continuous variation of phase difference between the LCP and RCP lights during longitudinal propagation, enabling the continuous transformation of the polarization state of the synthesized cylindrical vector fields along the longitudinal direction. In addition to LP light incidence, similarly, for circularly polarized illumination, the output light will be transformed into Bessel beams with customizable modes, with different Bessel beam cone angles for LCP light and RCP light. The light spots of the cylindrical vector fields rotate clockwise or counterclockwise around the optical axis according to the selection of the LCP and RCP cone angles. The metasurface is designed to provide a versatile solution for generating various vector optical fields with arbitrary mode combinations.

### 2.2 Method and theory

**Figure 2:**
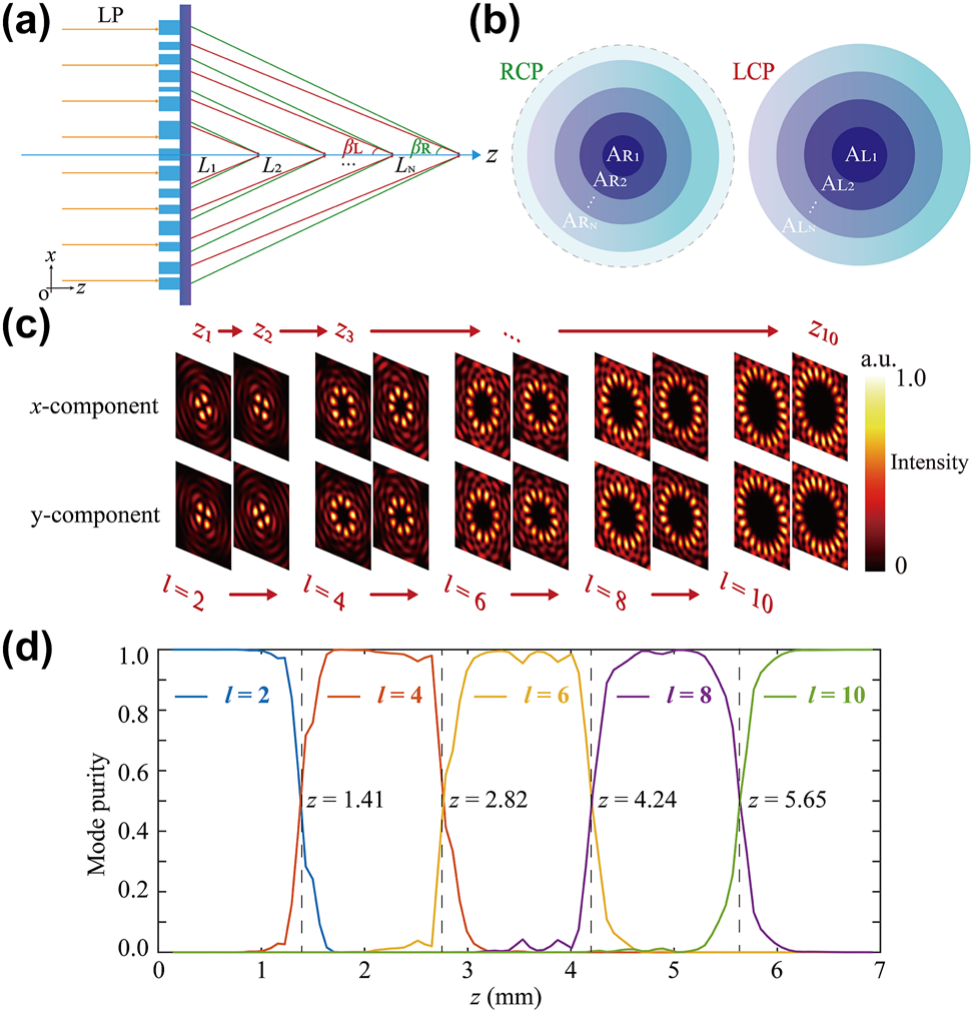
Principles for synthesizing longitudinally varying high-order vector fields. (a) Operating principle of the annular metasurface. The green and red lines represent LCP and RCP light, respectively. (b) LCP and RCP partition diagram of the metasurface. (c) Intensity distribution of the *x*-component and *y*-component in different regions under the *x*-LP light illumination. (d) Mode purity of synthetic vector optical fields.

Here, a more comprehensive explanation is provided regarding the methodology employed for the generation of high-order cylindrical vector fields and the variation in their respective orders. An LP light with an arbitrary polarization angle *θ* can be decomposed into two lights with opposite spin states, namely RCP and LCP. This decomposition can be described by the Jones vectors, expressed as follows:

(1) $$\left[\begin{array}{c} \cos\;\theta \\ \sin\;\theta \end{array}\right]=\frac{1}{2}\exp\left(-i\theta \right)\left[\begin{array}{c} 1\\ i \end{array}\right]+\frac{1}{2}\exp\left(i\theta \right)\left[\begin{array}{c} 1\\ -i \end{array}\right]$$

where [1, *i*]^*T*^ and [1, −*i*]^*T*^ stand the LCP and RCP components, respectively. The 2D radial vector optical field is generated when *θ* = *lφ*, where *l* is the topological charge and *φ* = tan^−1^(*y*/*x*) stands for the azimuth angle. In the synthesis of a 3D cylindrical vector optical field, the variation of *θ* with the propagation direction is imperative. Figure 2(a) illustrates that as the LP light incidents along the *z*-axis, the combination of RCP and LCP components introduces a variation in *θ* along the propagation direction, governed by distinct spatial phase factors (*k*_*R*_ *z* and *k*_*L*_ *z*), respectively. Correspondingly, different Bessel cone angles (*β*_*R*_ and *β*_*L*_) are required to enable different propagation constants of RCP and LCP as *k*_*R*/*L*_ = *k*_0_ cos *β*_*R*/*L*_, where *k*_0_ is the propagation constant in free space. Consequently, there will be a phase difference *γ* = (*k*_*R*_ − *k*_*L*_)*z* between the RCP and LCP components as *z* changes along the propagation direction. This allows for the synthesis of a longitudinally continuously varying high-order cylindrical vector field. Typically, generalized cylindrical vector fields can be separated into two eigenpolarization states, as depicted below:

(2) $$\begin{array}{ll} \left[\begin{array}{c} \cos\left(l\varphi +\frac{\gamma }{2}\right)\\ \sin\left(l\varphi +\frac{\gamma }{2}\right) \end{array}\right] & =\cos\left(\frac{\gamma }{2}\right)\left[\begin{array}{c} \cos l\varphi \\ \sin l\varphi \end{array}\right]-\sin\left(\frac{\gamma }{2}\right)\left[\begin{array}{c} \sin l\varphi \\ -\cos l\varphi \end{array}\right] \end{array}$$

where the case *l* > 1 corresponds to a higher-order cylindrical vector optical field. Evidently, the electric field vectors of the decomposed two eigenpolarization states are orthogonal to each other. The phase difference *γ* determines the intensity ratio between the two eigenpolarization states so that the output cylindrical vector fields with axisymmetric distribution exhibit continuity and periodicity during propagation. From Equation (2), the polarization transformation experiences a full period after the phase factor *γ* changes by 2*π*, the period over the propagation distance can be expressed as:

(3) $$\Gamma =\left|\frac{\lambda }{\cos\beta_{R}-\cos\beta_{L}}\right|$$

where *λ* is the wavelength in free space (see Section S1 of Supplementary Material for details of formula derivation).

Based on the analysis above, the phase distributions with LCP and RCP components at the plane *z* = 0 can be expressed as:

(4) $$\begin{array}{c} \phi_{R}\left(x,y\right)=l\varphi \left(x,y\right)+k_{0}\sin\beta_{R}\sqrt{x^{2}+y^{2}}\\ \phi_{L}\left(x,y\right)=-l\varphi \left(x,y\right)+k_{0}\sin\beta_{L}\sqrt{x^{2}+y^{2}} \end{array}$$

Under the incidence of RCP or LCP light, the metasurface generates the *l*th-order Bessel beams with varying cone angles of *β*_*R*_ and *β*_*L*_, respectively. When LP light is incident on the designed metasurface, the *l*th-order cylindrical vector optical field is synthesized.

Following phase modulation, incident light undergoes conversion into a Bessel–Gaussian beam. The aperture constraints of the phase mask limit the existence of the Bessel–Gaussian beam beyond the maximum propagation distance. This property is strategically exploited through the adoption of a spatially partitioned phase distribution design. The OAM topological charge is mapped onto concentric radial rings of the metasurface, resulting in the changes of topological charge along the optical axis. As depicted in Figure 2a, the ray-tracing method was employed, where *L*_*n*_ represents the central longitudinal length of the *n*th cylindrical vector optical field (*n* = 1, 2, …, *N*). This is achieved through the following approach, denoted as:

(5) $$\begin{array}{c} A_{R_{n}}=L_{n}\tan\beta_{R}\\ A_{L_{n}}=L_{n}\tan\beta_{L} \end{array}$$

where $A_{R_{n}}$ and $A_{L_{n}}$ represent the ring width of RCP and LCP light corresponding to the *n*th-order cylindrical vector field. The lengths of *L*_*n*_ are jointly determined by the radial length of the annular region $A_{R_{n}}$ and $A_{L_{n}}$ , along with the Bessel cone angle *β*_*R*_ and *β*_*L*_. Figure 2(b) provides an overhead perspective illustrating the division of the annular metasurface into regions for LCP and RCP light. The different cone angles in the design lead to variations in the sizes of these regions. Each region of them from inner to outer is labeled as $A_{R_{1}}$ , $A_{R_{2}}$ , …, $A_{R_{N}}$ , $A_{L_{1}}$ , $A_{L_{2}}$ , …, and $A_{L_{N}}$ . The areas of the annular metasurface for controlling the LCP and RCP are different, leading to intensity differences between them. Such differences can potentially influence the synthesis of the vector optical field. Comprehensive details can be found in Section S2 of Supplementary Material.

Next, 3D numerical simulations were conducted using the vectorial angular spectrum theory [53]. The simulations utilized LP light with a wavelength *λ* of 10.6 μm. cos *β*_*R*_ and cos *β*_*L*_ are set at 0.93 and 0.90, respectively. The equivalent numerical aperture of the metasurface can be obtained by the Equation (sin *β*_*R*_ + sin *β_L_*)/2, which controls the radius of the *l*-order Bessel beams [17]. The longitudinal simulation regions were extended to *z* = 7066.7 μm, and the metasurface was divided into 10 (2*N* = 10) annular regions, with the longitudinal length of each order set to 4Γ = 1413.3 μm. These sets allowed the calculation of the corresponding circular radius or ring radius for each region of the metasurface. These 10 annular regions of the designed metasurface control the generation of the five number of orders of cylindrical vector optical fields. The central circular regions $A_{R_{1}}$ and $A_{L_{1}}$ control the generation of the 2nd order, the 2nd ring-shaped regions $A_{R_{2}}$ and $A_{L_{2}}$ of LCP and RCP control the generation of the 4th order, and so on, with the outermost ring-shaped region $A_{R_{5}}$ and $A_{L_{5}}$ controlling the generation of the 10th order. The eigenpolarization states of the generated cylindrical vector optical fields with these even orders continuously transform and vary. In Figure 2(c), *x*-LP light is chosen as the incident light, and two traverse cross sections are selected with intervals of Γ/2 within each state in the region from *z*_1_ to *z*_10_ to observe the change in order. In each order, half of the first period was chosen as the initial plane, which means that *z*_1_ = 176.6 μm, *z*_2_ = 353.3 μm, *z*_3_ = 1590 μm, …, and finally *z*_10_ = 6006.7 μm. Observing the *x*-component and *y*-component, the cylindrical vector optical fields are transformed from the 2nd order to the 4th order and finally to the 10th order. This indicates that the scalar optical field has been successfully transformed into a 3D vector optical field. It is noticeable that the topological charge undergoes continuous rotation, and the number of focal spots in each region corresponds to the designated order. Moreover, the field distribution transforms at the boundary between the regions of two orders; see Section S3 of Supplementary Material for details.

The mode purities were derived through simulation employing the mode analysis method [54], as illustrated in Figure 2(d). The longitudinal length of each order of the vector optical field has been set to 4Γ. The results indicate that the mode purity of the 2nd-order vector optical field nearly reaches 1 within the range of z = 0 to z = 1.41 mm, while the mode purity of the remaining orders approaches 0. At the boundary between the 2nd and 4th-order vector optical fields, the mode purity of the 2nd-order vector optical field gradually decreases and rapidly converges to 0, while the mode purity of the 4th-order vector optical field fast approaches 1. Similarly, within the subsequent four regions, the mode purity of the respective orders tends toward 1, and the boundary exhibits a similar trend. Thus, the middle region of each order has a higher mode purity. This indicates that the crosstalk between different modes is effectively suppressed (see Section S4 of Supplementary Material for details).

### 2.3 Metasurface design

Based on the theoretical foundation, the composite-phase metasurface is chosen to obtain the desired phase distributions. A composite phase of Θ − 2*σ*Ω can be achieved on the cross-polarization component, where Θ and Ω represent the propagation phase and orientation angles of the unit cell separately, and *σ* = 1 or −1 represents the RCP or LCP incidence light, respectively. Consequently, the conjugate symmetry between the LCP and RCP components no longer exists, which leads to asymmetric PSOIs.

**Figure 3:**
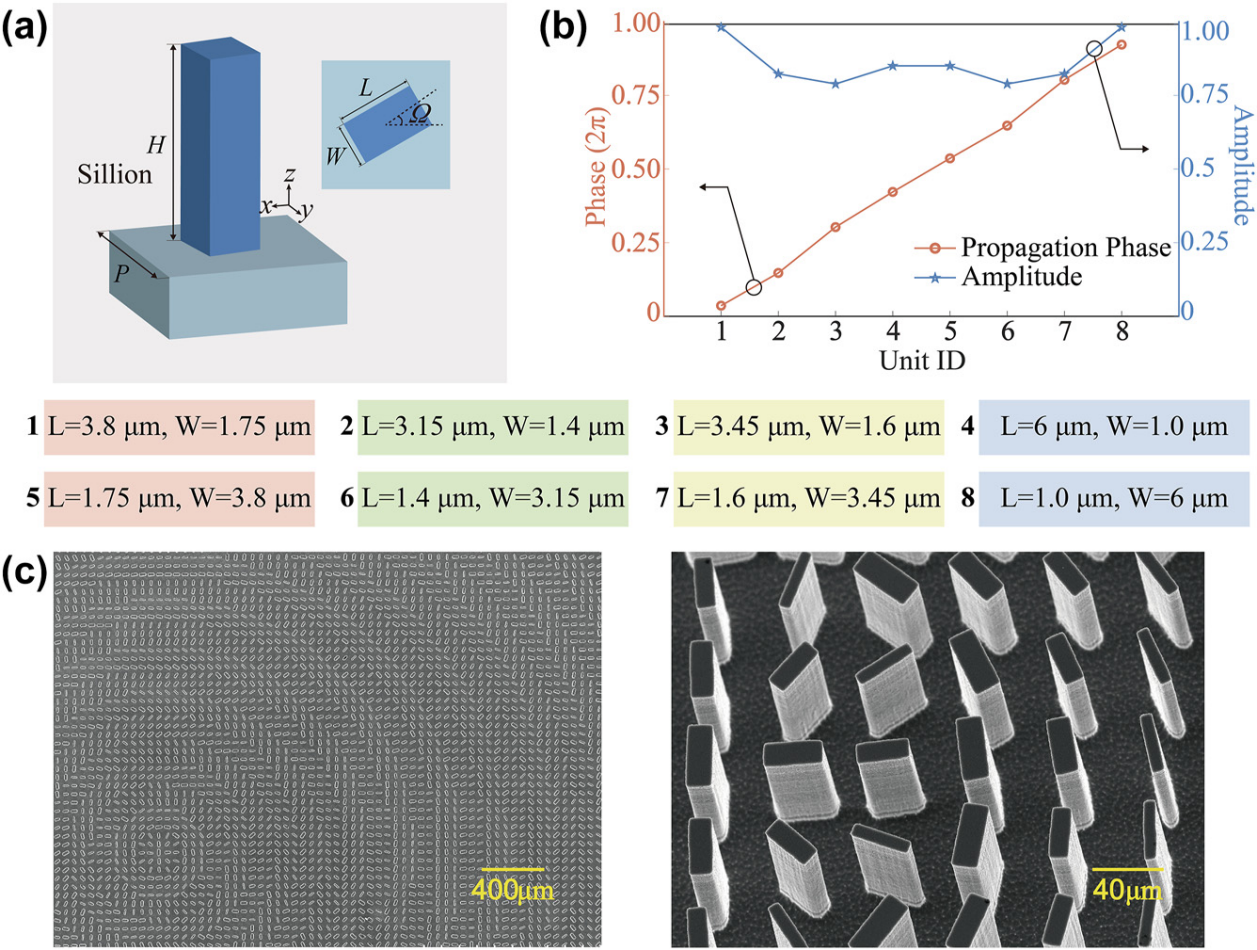
Schematic diagram of basic building blocks. (a) Schematic diagram of the unit cell, where *H* = 7.3 μm and *P* = 4.8 μm. The length *L* and width *W* of the eight nanorods are provided below. (b) Simulated propagation phase and polarization conversion amplitude of eight unit cells at a wavelength of 10.6 μm. (c) SEM images of a fabricated sample.

The schematic diagram of the unit cell is depicted in Figure 3. Each unit cell comprises a single layer of the silicon substrate and a silicon nanorod with a high refractive index, oriented at an angle Ω relative to the Cartesian coordinate system. This all-silicon structure has a constant height of *H*, but the dimensions, including length *L*, width *W*, and orientation angles Ω, vary. The unit cells are periodically distributed with a lattice constant of *P*, and the nanorods are at the center of each square substrate. The relevant unit cell schematic and the top-view diagram are illustrated in Figure 3(a). Eight unit cells are designed, each featuring an incremental propagation phase of approximately *π*/4. The polarization conversion amplitude and propagation phase are illustrated in Figure 3(b). Based on the required phase distribution of the metasurface, these eight unit cells are arranged discretely, and the required orientation Ω and propagation phase Θ of these unit cells that constitute the metasurface is determined as follows:

(6) $$\begin{array}{c} \Omega \left(x,y\right)=\frac{1}{4}\left[\varphi_{R}\left(x,y\right)-\varphi_{L}\left(x,y\right)\right]\\ \Theta \left(x,y\right)=\frac{1}{2}\left[\varphi_{R}\left(x,y\right)+\varphi_{L}\left(x,y\right)\right] \end{array}$$

Subsequently, a circular-shape all-silicon metasurface with a diameter of 6845 μm is fabricated by laser direct writing technology and inductively coupled plasma etching. Figure 3(c) depicts the scanning electron microscope (SEM) images of the proposed metasurface sample.

## 3 Experimental demonstration

**Figure 4:**
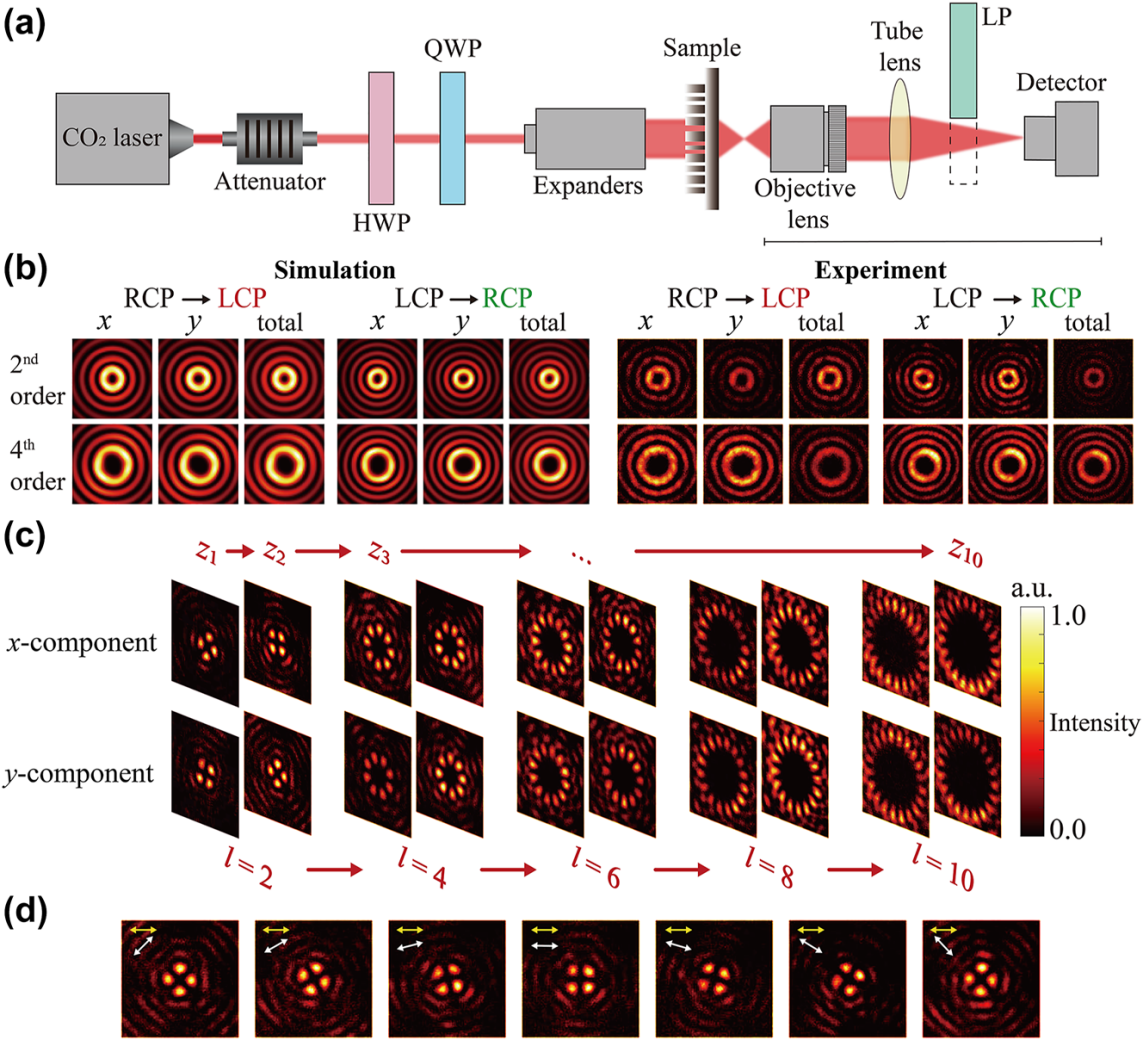
Experimental setup and results. (a) Schematic diagram of the optical characterization setup. The numerical aperture of the objective lens is about 0.55. (b) Simulation and experiment diagram of the 2nd and 4th order of *x*-component, *y*-component, and total intensity on the *x*–*y* plane of LCP and RCP light incidence. (c) Transverse plane images of the *x*-component and *y*-component at each region at the *x*-LP light incidence. (d) Intensity distributions of the transverse plane of the *x*-component of the 2nd-order cylindrical vector field at the *x*-LP incidence. The yellow arrow represents the polarization direction of the incident LP light, while the white arrow represents the orientation of the linear polarizer. QWP: quarter-wave plate; HWP: half-wave plate.

The schematic diagram of the optical setup for characterizations is depicted in Figure 4(a), in which a combination of a half-wave plate and a quarter-wave plate is placed in front of the metasurface to make the incident light the desired polarization state. Utilizing a beam expander (BXZ-10.6-1-3X) enlarges the beam diameter beyond the sample diameter. A linear polarizer positioned behind the sample is employed to acquire various components of the optical field. Figure 4(b) displays the simulated and experimental results with incident light set as LCP and RCP light individually. Only the 2nd and 4th-order cases are shown in this paper, presenting the distributions of the *x*, *y*-components, and total intensity on the *x*–*y* plane of the 2nd and 4th-order Bessel beams. The experimental results closely match the simulated. It can be observed that the optical spots have a ring-shaped (doughnut) distribution, and the ring-shaped intensity distribution continues to expand with increasing topological charge. This experimentally verifies that the output optical field has evolved into high-order Bessel beams. Additionally, the comparison between the ring width generated by LCP and RCP incidence reveals the difference in the Bessel beam cone angles for the incident LCP and RCP lights.

Figure 4(c) shows the intensity maps of the *x* and *y* components of each region at 10 transverse sections sampled between *z* = 176.6 μm and *z* = 6006.7 μm at *x*-LP light incidences. In agreement with the simulation results, the two planes selected in each order are spaced Γ/2 apart, and *z*_1_, *z*_2_, *z*_3_, …, and *z*_10_ represent the 1st, 2nd, 3rd …, and 10th cross sections selected, respectively. As *z* increases, the four centrally symmetric circular spots transform into 8, 12, 16, and 20 circular spots through intermediate states, with the central region of each spot remaining dark. This directly demonstrates that the generated optical fields have evolved into continuously tunable 3D high-order cylindrical vector optical fields, and the order of the cylindrical vector optical fields changes from the 2nd order to the 4th, 6th, 8th, and 10th order. In addition, the patterns of the *x* and *y* components of the light spots are always complementary.

To emphasize the continuous variation characteristic of vector optical fields along the longitudinal direction, a small interval longitudinal sampling was performed in our experiment. We used *x*-LP light to generate a 2nd-order cylindrical vector optical field, and then evenly divided it into seven slices with intervals of 60 μm, covering a distance of 360 μm. As shown in Figure 4(d), the intensity distribution of the 2nd-order cylindrical vector optical field continuously rotates as *z* changes, eventually coinciding with the first one. This indicates that the optical field undergoes almost two cycles and nearly agrees with the initial spot distribution. More field distributions on the *xz* plane along the propagation direction are shown in Section S5 of Supplementary Material. In addition to these observations, the average diffraction efficiency of the metasurface at both LCP and RCP incidence is measured, reaching 86 %. Here, the diffraction efficiency is defined as the ratio of the power of the deflected light to the total power of the output light (see Section S6 of Supplementary Material for details).

## 4 Conclusions

In conclusion, we proposed a novel approach of spin-decoupling spatial partitioning to generate complex 3D vector optical fields along the propagation direction. By exploiting the asymmetry PSOIs, the regional displacement design of two orthogonal polarization states enables arbitrary optical field control in 3D space. As a proof-of-concept demonstration, the experimental verification for the generation of longitudinally varying high-order cylindrical vector optical fields is provided. The observed modes ranged from the 2nd to the 10th order along the propagation direction at the linear polarization incidence, while circularly polarized incidence resulted in the generation of high-order Bessel beams. This work not only validates the feasibility of our approach but also opens up avenues for broader applications of pillar-symmetric 3D vector optical fields. The potential extends to more complex vector optical fields, including the full Poincaré vector optical field, vector optical fields with multiple singularities, and nonpillar-symmetric vector optical fields. These advancements hold promise for applications across diverse domains, such as biophotonics, quantum optics, and communications.

## Supplementary Material

Supplementary Material Details
