# Supplementary material for: Longitudinally continuous varying high-order cylindrical vector fields enabled by spin-decoupled metasurfaces
Source: Nanophotonics. 2024 Mar 4;13(9):1657–64. doi: 10.1515/nanoph-2024-0008 (PMC11636513; doi:10.1515/nanoph-2024-0008)
Supplement: Supplementary file 1 — Supplementary Material Details [file j_nanoph-2024-0008_suppl_001.docx]

**Supplementary Material**

**Longitudinally continuous varying high-order cylindrical vector fields enabled by spin-decoupled metasurfaces**

Xinye He, Hanlin Bao, Fei Zhang, Tongtong Kang, Mingbo Pu, Yan Chen, Yinghui Guo, Jintao Gong, Mingfeng Xu and Xiangang Luo*

**S1. Derivation of periods**

The phase difference *γ* between the RCP and LCP light along the propagation direction is given by:

$$\gamma=(k_{R}-k_{L})z$$

where $k_{R}=k_{0}\cos\beta_{R}$, $k_{L}=k_{0}\cos\beta_{L}$ and $k_{0}=\frac{2\pi}{\lambda}$, thus Equation (S1) can be rewritten as:

$$\gamma=\frac{2\pi(\text{cos}\beta_{R}-\text{cos}\beta_{L})z}{\lambda}$$

When the phase difference *γ* between the LCP and RCP light is 2π, the synthesized polarization state completes a 180° rotation, indicating a full period *Γ* of the polarization transformation. Based on Equation (S2), the period over the propagation distance is expressed as:

$$\Gamma=\frac{2\pi\lambda}{2\pi\text{(cos}\beta_{R}-\text{cos}\beta_{L})}=\frac{\lambda}{\text{cos}\beta_{R}-\text{cos}\beta_{L}}$$

**S2. Influence of intensity differences between LCP and RCP on the vector optical fields**

The annular metasurface areas designed for controlling LCP and RCP lights differ, resulting in different intensities for each. Simulations are conducted to generate the vector optical field, denoted as *E*^i^, under conditions where the regions of LCP and RCP light have identical intensity distributions. Similarly, the electric field *E*^s^ is computed under conditions where these regions exhibit different intensity distributions. The simulated electric fields *E*^i^ and *E*^s^ can be approximated as follows^[54, 55]^:

$$\begin{matrix} E^{i}(x,y,z)=\frac{\sqrt{2}}{2}E^{L}+\frac{\sqrt{2}}{2}E^{R} \\ E^{s}(x,y,z)=\sqrt{\alpha}E^{L}+\sqrt{1-\alpha}E^{R} \end{matrix}$$

where *E*^L^ and *E*^R^ represent the LCP and RCP Bessel optical fields, respectively, and the α represents the intensity ratio of LCP in the entire optical field. The similarity between *E*^i^ and *E*^s^ can be expressed by^[56]^

$$V=\frac{|\langle E^{i}(x,y,z)|E^{s}(x,y,z)\rangle|^{2}}{|\langle E^{i}(x,y,z)|E^{i}(x,y,z)\rangle|^{2}}$$

When a difference exists in the intensity ratio between LCP and RCP light, the variation in similarity between the generated optical field and the desired optical field is illustrated in Figure S1.

**
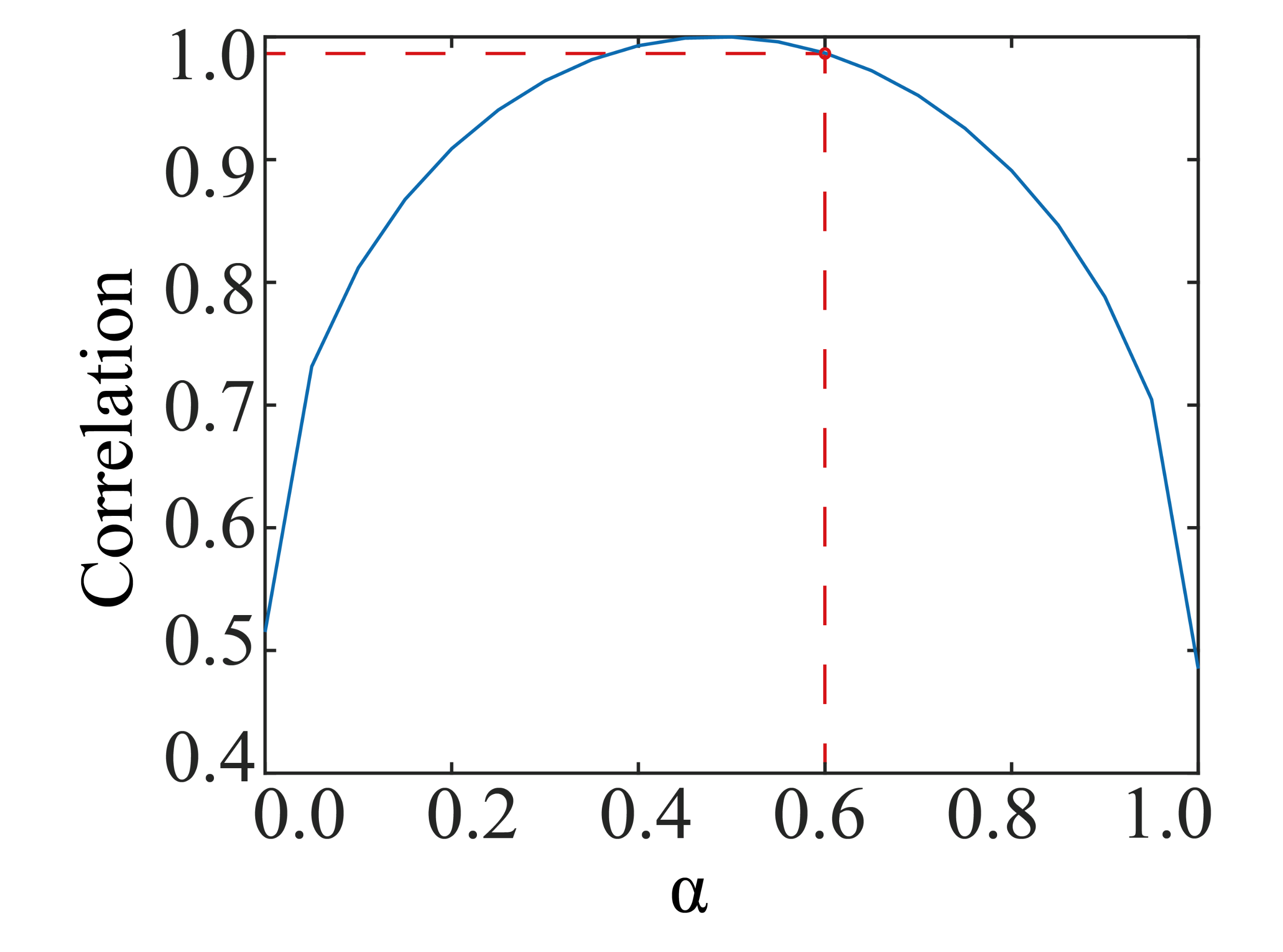
**

**Figure. S1:** Effect of intensity differences between LCP and RCP on the vector optical fields.

From Figure S1, it can be observed that when there is a difference in the intensity ratio between LCP and RCP light, it results in partial distortion of the vector optical field. However, in our designed metasurface, the area ratio between LCP rings and RCP rings leads to an intensity ratio approximately in the range of 0.6:0.4, and the similarity of the vector optical field remains above 95%.

**S3. Field transformation at the boundary of two different orders**

Figure S2 shows the simulated electric field distributions on the *xy*-plane under *x*-polarized light incidence, presenting the *x*-component and *y*-component of the boundary region of the two orders. The chosen longitudinal region is centered at the boundary point of two orders, denoted as *Z_t_* (*t* = 1,2,3,4), and sampled at intervals of 30 µm, spanning a range of 180 µm from *Z_t_* - 90 µm to *Z_t_* + 90 µm. Here, *Z*_1_ = 1.41 mm, *Z*₂=2.82 mm, *Z*₃=4.24 mm, and *Z*₄=5.65 mm. A gradual and continuous transformation of the vector optical field from one order to another can be observed within the boundary regions.


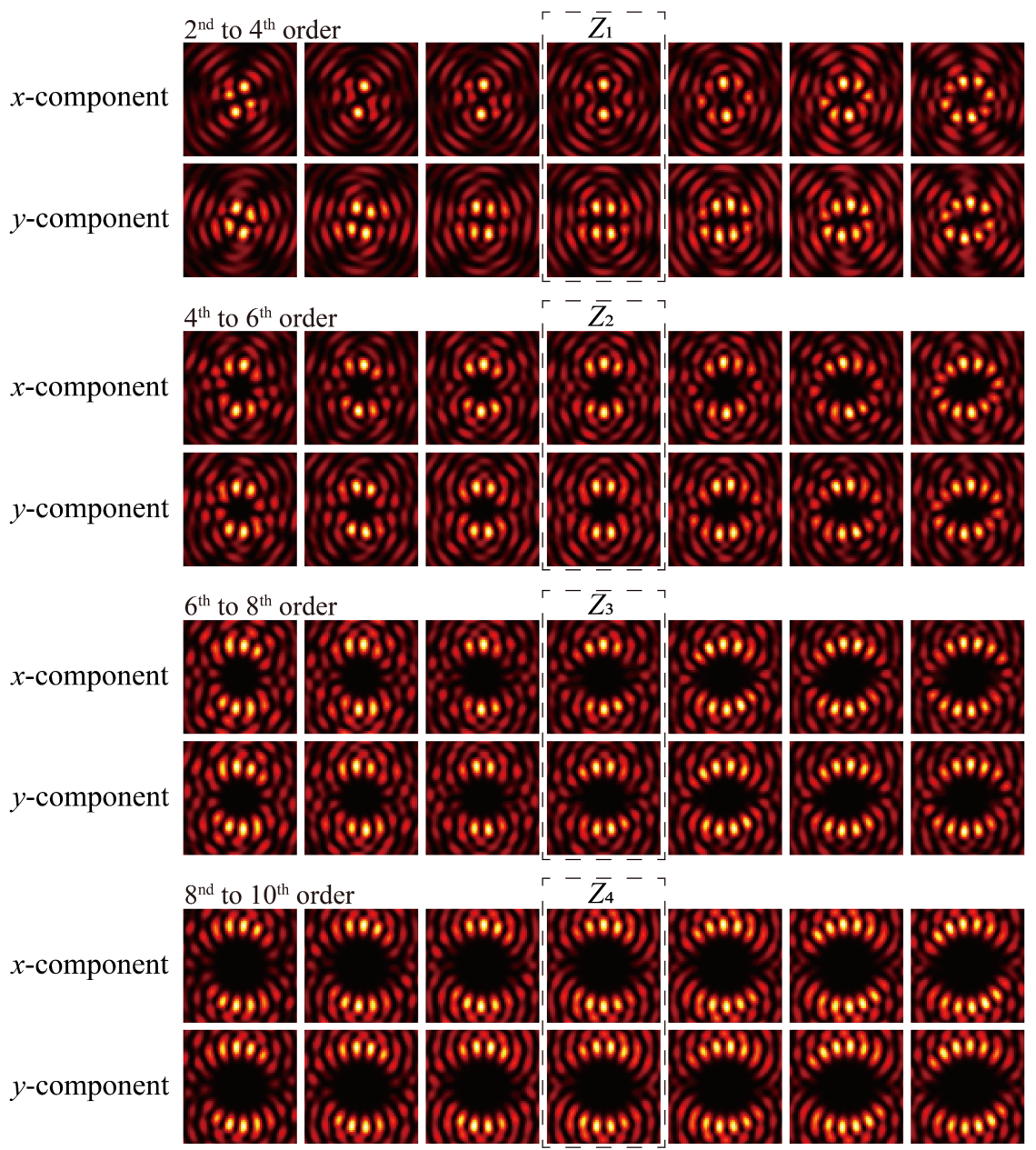


**Figure. S2:** Changes in the boundary region of the two orders. The electric field distributions in the boundary region are shown for the 2^nd^ to 4^th^, 4^th^ to 6^th^, 6^th^ to 8^th^, and 8^th^ to 10^th^ orders, respectively. Boxed are the boundary points of two orders.

**S4. Mode purity analysis of the vector optical fields**

To comprehensively assess the crosstalk between the superimposed vector optical fields, we simulate the vector optical field under the scenario where no rings were partitioned, denoted as the ideal optical field *E_l_^d^*. By computing the inner product between the ideal optical field and the actual generated optical field in the paraxial region (the paraxial region is circular in shape, with a radius three times larger than that of the 10^th^-order Bessel beam, measuring about 162 µm), the mode purity of the actual generated optical field relative to the ideal optical field is obtained by:

$$v_{l}(z)=\frac{|\langle E_{l}^{d}|E(x,y,z)\rangle|^{2}}{|\langle E_{l}^{d}|E_{l}^{d}\rangle|^{2}}$$

Similarly, the normalization is conducted:

$$V_{l}(z)=v_{l}(z)/\sum_{l=0}^{\infty} v_{l}(z)$$

The generated vector optical field can be regarded as the composed of a series of LCP and RCP lights carrying different topological charges, which can be expressed as^[1]^:

where *J_l_* represents the *l*-order Bessel function and $r=\sqrt{x^{2}+y^{2}}$. To analyze the crosstalk between different modes, the expansion coefficients $c_{l}^{R}$ and $c_{l}^{L}$ of LCP and RCP components of the generated vector optical field are separately examined:

$$\begin{matrix} c_{l}^{R}=\langle E^{R}|E(x,y,z)\rangle=\int E^{R*}\cdot E(x,y,z)\text{d}S \\ c_{l}^{L}=\langle E^{L}|E(x,y,z)\rangle=\int E^{L*}\cdot E(x,y,z)\text{d}S \end{matrix}$$

Then, the normalization is conducted:

$$C_{l}^{R/L}=c_{l}^{R/L}/\sum_{l=0}^{\infty} [c_{l}^{R}+c_{l}^{L}]$$

The expansion coefficients for LCP and RCP are displayed in Figure S3, and the results show that the expansion coefficients of the respective orders of the LCP lamps and the RCP are about 0.5 in the corresponding regions, with a rapid transition of the topological charge in the junction region and close to zero in the other regions.


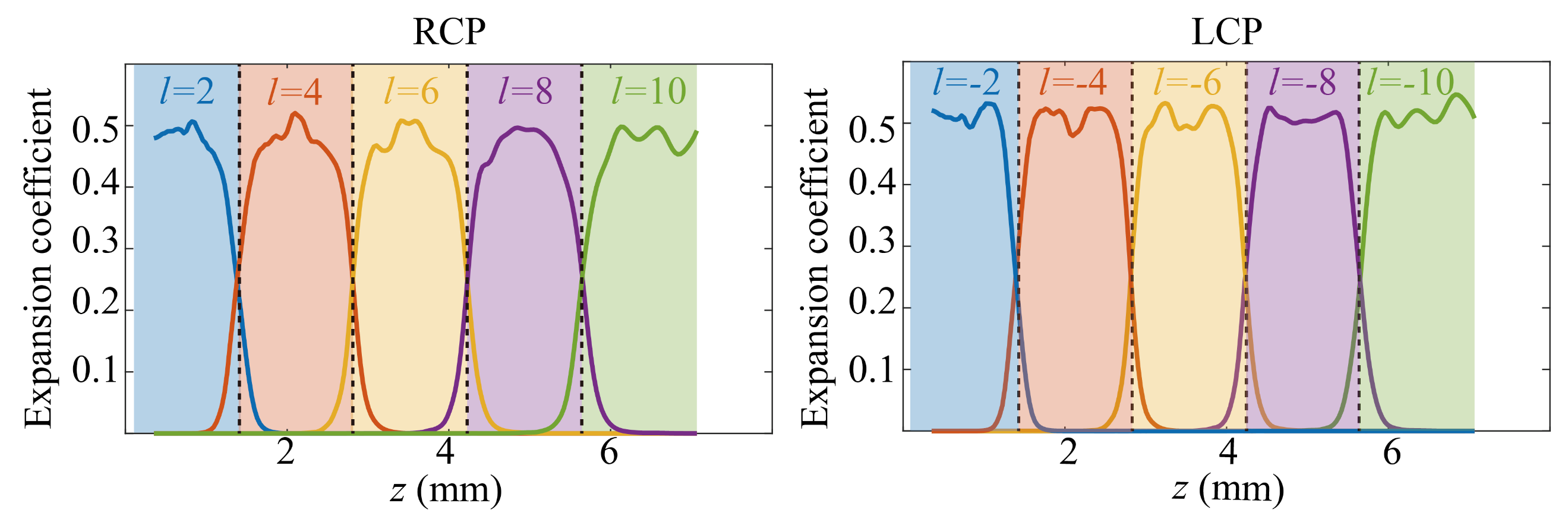


**Figure. S3:** Variation of expansion coefficients of RCP and LCP with propagation direction.

**S5. Longitudinal continuous variation of the vector optical fields**

Figure S4 (a) presents supplementary illustrations depicting experimental results for each order of the vector optical field, longitudinally sampled at small intervals along the longitudinal direction. Cylindrical vector optical fields of orders 2, 4, 6, 8, and 10 are generated under the illumination of *x*-LP light. For each order, a longitudinal length was selected and uniformly divided into seven slices, spaced 60 μm apart and covering a total of 360 μm—approximating the distance of one period, denoted as *Γ*. Notably, it can be observed that the optical spot of each order is rotated to coincide with the initial spot. To further demonstrate the continuous variation of the vector optical field across the entire effective longitudinal length, Figure S4 (b) shows the simulated intensity distribution of the cylindrical vector optical field in the *xz* plane along the propagation direction. The *x*-component is observed to be complementary to the *y*-component focal spot at the same *z*-section. A total of 16 spots are identified over the entire longitudinal region, suggesting that each section of the region corresponds to 4 periods. Additionally, the total intensity exhibits continuous variation across the entire longitudinal region.


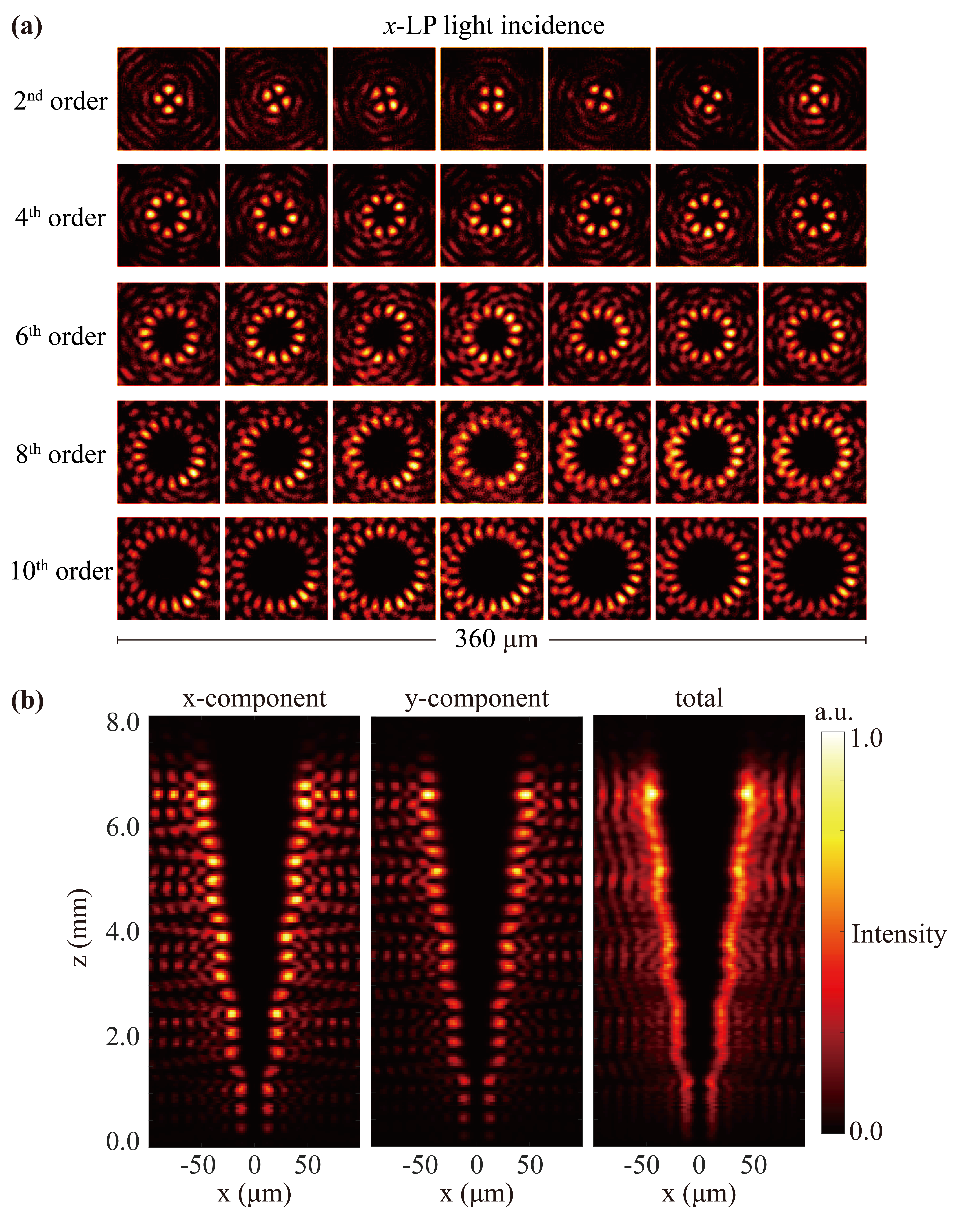


**Figure. S4:** Longitudinal continuous variation of the vector optical fields. (a) Small interval sampling of cylindrical vector optical fields of orders 2, 4, 6, 8, and 10 under the *x*-LP light illumination. (b) Simulation of the longitudinal distribution of the *x*-component, *y*-component, and total intensity for the *xz*-plane.

**S6. Measurement of average diffraction efficiency**

Firstly, a 10.6μm CO_2_ laser served as the light source to illuminate the edge position of the metasurface, while a power sensor was positioned near the metasurface to measure the output light intensity. Subsequently, the power sensor was repositioned to an angle of (β_R_+β_L_)/2 to assess the light intensity after deflection. Given the proximity of the light spots after LCP and RCP light deflection, all can be captured by the powermeter. Thus, when linearly polarized light was used for incidence, the average diffraction efficiency is measured by the ratio of the power of the deflected light to the total power of the output light.


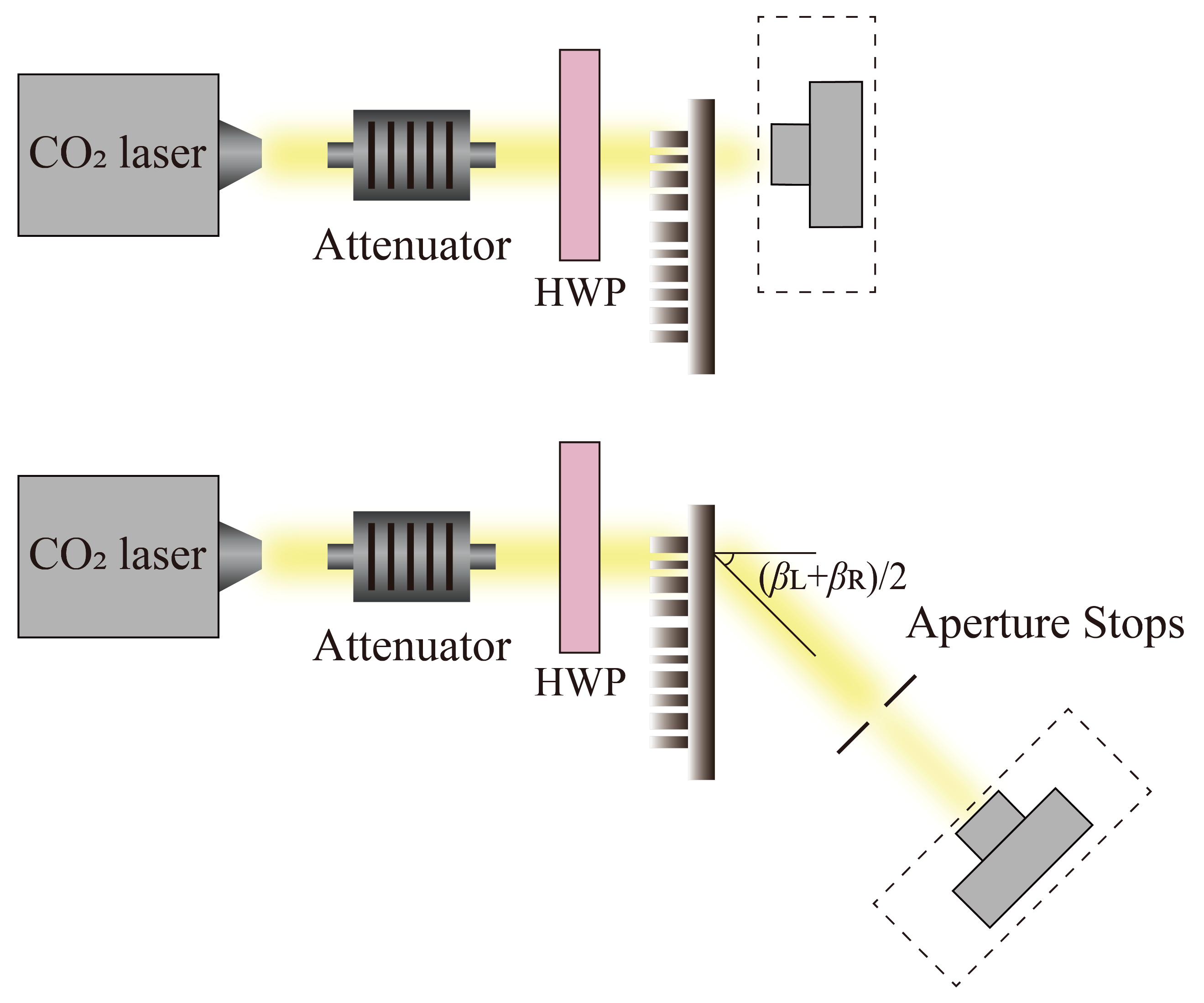


**Figure. S5:** Schematic diagram of the optical characterization setup of measuring average diffraction efficiency.

**References**

1. A. Forbes, M. de Oliveira, and M. R. Dennis, "Structured light," Nat. Photonics, vol. 15, no. 4, pp. 253-262, 2021.
2. M. Mansouree, A. McClung, S. Samudrala, and A. Arbabi, "Large-scale parametrized metasurface design using adjoint optimization," ACS Photonics, vol. 8, no. 2, pp. 455-463, 2021.
